# Social-ecological considerations informing a universal screening strategy for sleep health in the community

**DOI:** 10.3389/fpsyt.2023.857717

**Published:** 2023-03-20

**Authors:** Sarah Blunden, William McKellin, Thomas Herdin, Osman S. Ipsiroglu

**Affiliations:** ^1^Appleton Institute of Behavioral Science, Sleep and Circadian Group, Central Queensland University, Wayville, SA, Australia; ^2^Department of Anthropology, University of British Columbia, Vancouver, BC, Canada; ^3^Division of Transcultural Communication, Department of Communication Studies, Paris-Lodron-University Salzburg, Salzburg, Austria; ^4^H-Behaviors Research Lab (Previously Sleep/Wake-Behaviors Lab), BC Children’s Hospital Research Institute, Vancouver, BC, Canada; ^5^Division of Developmental Pediatrics, Respirology, and Child and Adolescent Psychiatry, Department of Pediatrics and Psychiatry, Faculty of Medicine, The University of British Columbia, Vancouver, BC, Canada

**Keywords:** community health, multi-professional team, public health priority, iatrogenic harm, medical anthropology

## Abstract

“Poor sleep health” (PSH), defined as reduced amount of sleep and non-restorative sleep, affects cognitive, social and emotional development. Evidence suggests an association of sleep deprivation and mental health problems; however, there are no universal concepts allowing a first-tier screening of PSH at a community level. The focus of this narrative review is to highlight the cultural context of the current medicalized approach to PSH and to suggest social ecological strategies informing new and holistic community-based screening concepts. We present two conceptual screening frameworks; a “medical” and a merged “social emotional wellbeing framework” and combine them utilizing the concept of “ecologies.” The first framework proposes the incorporation of “sleep” in the interpretation of “vigilance” and “inappropriate” labeled behaviors. In the first framework, we provide a logic model for screening the myriad of presentations and possible root causes of sleep disturbances as a tool to assess daytime behaviors in context with PSH. In the second framework, we provide evidence that informs screening for “social emotional wellbeing” in the context of predictive factors, perpetuating factors and predispositions through different cultural perspectives. The distinct goals of both frameworks are to overcome training-biased unidirectional thinking and *a priori* medicalization of challenging, disruptive and/or disobedient behaviors. The latter has been explicitly informed by the critical discourse on colonization and its consequences, spearheaded by First Nations. Our “transcultural, transdisciplinary and transdiagnostic screening framework” may serve as a starting point from which adaptations of medical models could be developed to suit the purposes of holistic screening, diagnosis, and treatment of complex childhood presentations in different cultural contexts.

## Sleep health and the need for a community based screening concept

*Sleep* is essential for one’s health and well-being and is at a lifetime maximum during childhood. Conversely, poor sleep health (PSH), defined as a reduced amount of sleep and poor quality of non-restorative sleep, are increasingly common in modern society. Insufficient and/or suboptimal sleep is an emerging public health issue (1). Large epidemiological studies consistently show an association between sleep quality and duration with physiological health outcomes, such as obesity, diabetes, and cardiovascular disease (2) the foundations of which have been recognized in childhood. In the neuropsychological domain, sleep is also essential for academic performance, with meta-analyses confirming the detrimental impact of reduced sleep quality and duration on the ability to learn and subsequently impair academic performance in children and adolescents (3). PSH is a major contributor to reduced attention span (4), as well as slower reaction times, difficulty learning and consolidating memory, reduced capacity to emotionally and physically self-regulate, hyperactivity, risk-taking, and even aggression. It is worth noting that improving sleep health has a positive effect on all of these domains (5–7).

### Sleep disturbances and mental health

Furthermore, neurodevelopmental disorders and mental health disorders are associated with a high prevalence of sleep disturbances and disorders. In the past decade, the prevalence for neurodevelopmental and mental health disorders has increased. For example, the most widespread neurodevelopmental disorder is attention deficit hyperactivity disorders (ADHD) which globally affects 7.2% of children and adolescents under the age of 18, and 2.5% of adults (8–10); however, depending on where the study has been conducted, the rates for adults may reach up to 8.1% (11) and 4.4% (12). Similarly, prevalence rates of autism spectrum disorder (ASD) fluctuate between 3.0–11.6% in Europe, and 1.6–18.9% in Asia, respectively, while data from many countries and/or continents, which may not offer nationwide sub-specialized medicine, are missing, e.g., Africa (13, 14).

### A transcultural, transdisciplinary, and transdiagnostic approach for a community-based screening concept

Families often conceptualize their child’s development, academic difficulties, and poor learning outcomes as medical issues and visit health care professionals for guidance. Thus, poor attention focus and related “*challenging, disruptive and/or disobedient behaviors*” and related concerns are the “medicalized” main complaints (15). However, with the increasing recognition of sleep as the modulating factor of health, there is a need to review sleep related concerns from a community-based and public health perspective. Moreover, publicly-funded pediatric sleep services are still not universally accessible across most high-income countries, such as Australia, Austria, and Canada, and the applicable sleep medicine related information often exceeds available second- or third-tier service-based knowledge (16). These shortcomings have been exacerbated by the COVID-19 pandemic, which led to disruptions in healthcare delivery and worsening mental and sleep health.

Screening for PSH offered at the community level is a strategy to leverage opportunities for early interventions, thus avoiding iatrogenic harm during unduly long wait times. As a first step to revise our current subspecialty-driven sleep medicine practices, we reviewed our traditional modes of service delivery with the goal to introduce a transcultural, transdisciplinary and transdiagnostic approach and to address PSH related concerns in context with basic recognizable patterns of daytime tiredness, affected wellbeing and restlessness at day and night time (16, 17).

In this concept paper, we are justifying the medical and socio-ecological background of this screening concept and review how transcultural, transdisciplinary, and transdiagnostic thinking may support the creation of an individualized, tailored assessment, and intervention framework.

## The ecological aspects in sleep health: An overview

*The concept of ecology* has influenced Western culture for some time. The Oxford Dictionary defines ecology as the branch of biology that deals with the relations of organisms to one another and to their physical surroundings (18). Ecological theories such as Bronfenbrenner’s ecological model (19) provide a framework from which observation and exploration can be used to understand the context of an individual’s distinct development and interactions at various levels over time. Indeed, when conceptualizing health, based on the contributing factors to the health of the individual, and community, all aspects of social and emotional well-being must be taken into account and exploration of inequities must be considered from a position of *Cultural Humility* with ongoing reflective practices and an awareness of power imbalances (20). In the context of sleep health for children the integration of ecology is an opportunity to close existing gaps in the following aspects:

### Child and family centeredness

The core question is how to best assist the needs of families and children with PSH in the community. As an example, in the context of ADHD, inattention, short- or long-term lack of focus and hypermotor-restlessness at day and/or night-time can have many etiologies and parents may describe these symptoms in their children’s lives and in *their* terms (21, 22). Exploring daytime behaviors in association with PSH, in collaboration with the affected individual allows for a more *mutually* shared inclusive approach to behavioral sleep- and wake-medicine adding to the clinical gaze. Similar to the work of an athlete with a trainer or coach, understanding the *lived experiences* more in-depth provides insight into the predictive and perpetuating factors that contribute to their predispositions and dispositions (23). Predictive and perpetuating factors include multiple elements that are not restricted to physiological etiologies, but also include psychological, environmental, familial, psychosocial, and potentially genetic or epigenetic factors. Consequently, treatment becomes not only more individualized for these children, but also more resourceful and efficient, as it considers the crucial areas affecting cognitive and social–emotional development, behavior, and general wellbeing, generally defined as judging life positively (24, 25). The ongoing discussion on how to implement individually meaningful outcome measures in daily practice is the advanced result of this discourse (26).

### “Medicalized” vs. “observation-based” semiotics

*The term* “*attention*” is associated with “*performance against* an *a priori standard*,” whereas the term “*vigilance*” (27) allows the notion of self-determined “*sustained attention*” and “*state of concentration on reaching an aim*”—a concept, which is from an ecological perspective more natural or ecologic. The medicalization of the initially observation-based descriptive vigilance concept has resulted in a variety of lab-based tests for its evaluation, such as the Mackworth clock test in 1948 (28–31). However, undertaking such vigilance tests is boring and they do not actively invite participation or attention, particularly not for children and adolescents (32). Therefore motivation, sleepiness, and capacity to attend may be compounding factors on the lab-based assessment of vigilance. Therefore, we suggest revisiting the concept of vigilance and using it in the Head suggested way: “*sustained attention*” and “*state of concentration on reaching an aim*.” Similarly, the term “*restlessness*” has been medicalized and is associated by parents and professionals mainly with hypermotor-restlessness at daytime and might be missed as a cause of sleep disturbances if not explicitly explored (33). However, as daytime restlessness often presents jointly with nighttime restlessness and results in PSH, which again perpetuates daytime restlessness and cognitive and behavioral dysfunction (16), the exploration of observation-based nighttime restlessness (e.g., during falling asleep and in sleep) is crucial (16, 17).

Together, the observation-based descriptions of “*vigilance*” and “*hypermotor-restlessness during day and night time*,” offer a novel conceptual observation-based exploratory framework to understand “*dysregulation*” or “*challenging, disruptive and/or disobedient*” sleep- and wake-behaviors (16, 32).

### Pharmacological vs. collaborative “social ecology-informed” approach. The need for a collaborative approach is readily apparent in the care for children with ADHD

When seeking treatment for their child, parents of children living with ADHD report a positive effect of community-based support (34). Community-based support such as navigation help or coaching, contributes to the resilience of the family and highlights the importance of assistance and collaborative work to implement interventions *with* families rather than *on* families. We can subsume that the individual child’s and family’s experience must be understood in its community context or social ecology. In behavioral medicine, e.g., for the treatment of ADHD, (cognitive) behavioral therapy has already been developed and evaluated, and is recommended as a first line measure (Subcommittee on Attention-Deficit/Hyperactivity Disorder) (7). However, it is time-consuming and requires involved parties to adopt the understanding of and therefore, lived culture to accept the recommendations (21). The challenge in embarking in this process might explain the upward trend in drug prescriptions, despite the fact that individual physicians are often not convinced of medication effects and/or see medication practices as controversial (21, 35, 36). This trend, without adequate investigation of broader predictive and perpetuating factors, such as family culture and biopsychosocial factors or PSH, reflects an imminent crisis, which builds on solely medication based strategies (35, 37–39). In the context of the COVID-19 pandemic, this trend has already become reality and raises further concerns regarding medication focused interventions (40).

### Technology vs. patient as co-participant approach

A major critique in Western medicine and medical training is their narrow focus (41, 42). Historically, the modern, medication-, or technology-centered medicine that we have all grown up with, was built on an in-depth cause-and-effect investigation (single-cause-and-single-effect). Modern medicine, with its foundation in autopsy research approach (i.e., focusing on anatomy and pathology), has been instrumental in creating the contemporary discourse of cause-and-effect-interactions, thus opening the floor for in-depth phenotyping and overcoming the concept of broad hermeneutic interpretations as Foucault describes “*In The Birth of the Clinic: An Archeology of Medical Perception*” (41). Therefore, similar to medications, which we perceive as a “fixing” strategy, data collection that describes symptoms with modern technology, e.g., electrophysiological information has been very much appreciated and thus, has become more prioritized over time. In sleep medicine, this resulted in polysomnography (PSG) focus to the detriment of a deeper discourse about other predisposing and contributing factors.

The modern technology-centered approach enforces the generation of model situations, which are often far from the reality of the lived experiences of individuals or even communities (25). Time and financial constraints in modern day clinical psychiatric practice compound these problems. As clinical scientists dealing with sleep issues, we see every day the restrictions that current clinical sleep health concepts reveal (25, 33, 43, 44). The limited success of sleep health campaigns, e.g., in school settings, might also be explained by their focus on discipline specific professional perspectives and their inability to resonate with lived experiences (43). In consequence, there is a need to transform the patient-as-object in examinations into a co-participant in care through effective co-constructed communication, interaction, and goal setting (25, 45)

### Reciprocal communication to overcome boundaries of the norm

There is a need for concepts that support what the individually tailored assessment of what parents/caregivers or professionals in the medical or educational system see and define as the “norm” and/or exceeding the norm and how this informs patient/care provider interaction and goal setting. Whereas the various understandings of “norms,” are based on the very specific, individual background, education, and training culture in other words one’s individual culture (23, 46, 47). The medical model is underpinned by a historical power discrepancy between the patient and medical professionals. The resulting power gradient fosters a paternalistic communication in a medico-centric model of care, e.g., the typical Anglo-American communication style, addressing patients with their first names, violating natural boundaries, and affecting goal setting and outcomes. Conversely, a patient-centered communication approach advocates for a reciprocal co-constructed patient-doctor understanding that does not need to fit into the “norm” (48) (pg. 744).

Reciprocal communication is very specific to each situation uniting the dimensions of communication and culture. We live in a symbolic world, shaped by culture. Clifford Geertz (1973, p. 89) (49) understands the concept of culture as symbols, knowledge, and attitudes, and defines culture as “*an historically transmitted pattern of meanings embodied in symbols, a system of inherited conceptions expressed in symbolic forms by means of which men communicate, perpetuate, and develop their knowledge about and attitudes toward life*.” Thus, an individual has a personal identity (individuated self) as well as a sociocultural identity which includes ethnic, cultural, religious, spiritual, gender, age, relational, and other role conceptions (50). Recognizing the symbolic world of the other is especially important in mental health services where explorative and person centric interviewing are fundamental in opening new pathways to communication beyond the boundaries of the “norm.”

An example of this is neglect of restless legs syndrome in vulnerable children is a modern parable for systemic errors in communication. Restless legs syndrome (RLS), is sensorimotor neurologic disorder causing PSH due to discomfort/pain urging to movements mainly of the legs. RLS is a well-recognized condition in pediatric and adult medicine. However, because the traditional diagnostic criteria are based on patient reported symptoms, RLS has been missed in children with neurodevelopmental conditions or mental health disorders until 2013, when the diagnostic criteria were expanded by descriptions obtained through behavioral observations (51) and reciprocal communication in history taking (25, 52). Another example of missed causes of PSH is the conundrum of restless sleep disorder (RSD), a major complaint of parents, for which we did not have an answer until 2018, when DelRosso and colleagues combined clinical in-depth observations and exploration of individual parental descriptions in junction with technical medicine (53, 54).

## Integrating a medical and socio-ecologic framework in sleep assessments for children

### The inclusive collaborative approach

In an attempt to overcome the traditional medicalized approach, we, as a large group of clinicians, in dialogue with Indigenous and non-Indigenous community-based partners, reviewed from a broader perspective why sleep health has not been recognized as a public health emergency and what is needed to implement a community based screening for PSH. Because of their wider holistic view of health and historical experiences of suffering and trauma, Canadian and Australian Indigenous models of health perception have informed and framed our clinical understanding. We are presenting first a medical logic model for screening PSH related causes and working out first line measures, and second how this can be implemented in the shared ecology and of experiences and environment, thus not perpetuating e factors within any presenting health problem.

### The medical logic model, strengths, and limitations

**Figure 1:**
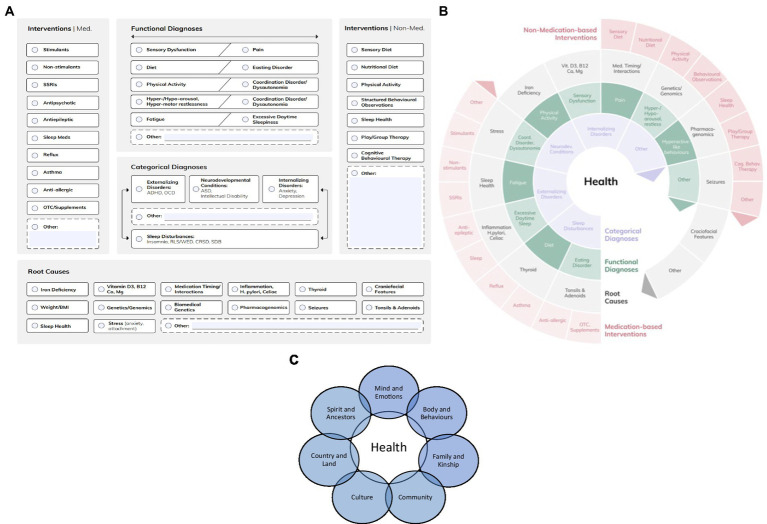
**(A)** The initial Medical Logic Model (16). **(B)** The Medical Logic Model in a wheel format for highlighting the changing and not yet explored interconnections with the permission of Kleanthes Publishers (57). **(C)** SEWB in Indigenous Australians. Adapted from SEWB framework (58).

First, we agreed that a visualized medically informed screening framework was of the utmost importance for making our clinical knowledge transparent and available. This model aims to overcome compartmentalization in the communities as well as in academic settings (16, 55, 56). Given the complexity of individual biopsychosocial circumstances, the first step was the re-set of categorical day and nighttime-related diagnoses (e.g., ADHD and insomnia, respectively) in equal relation. Combining them with functional diagnoses, which are usually observed first by parents/caregivers and allied health care professionals (e.g., vigilance, hyper-motor-restlessness, sensory dysfunctions, pain, and fatigue), and probable predictive factors recognized by the members of multi-professional community or university based teams was the next step. Figure 1A is a visual representation depicting the integration of categorical and functional diagnoses, taking into account etiologies and root causes. Figure 1B visualizes the dynamic interconnections between these areas.

### Expanding the medical model by social ecology

To become a learning system, exploration of the culture of the patient/parents/caregivers has been suggested as an integral part of complex assessments as early as in the 1960s (59). In the context of a community-based screening concept for PSH in children, acknowledging the lived experience and ecology of the community is a navigational aid to expand and complement the medical paradigm. Here, the concept of social ecology might be the one offering a pragmatic solution, overcoming cultural barriers and supporting the investigation of root causes with a shared language based on observation and exploration—a form of “*transdisciplinary health”* approach suggested by Assmuth et al. (60). Arguably, in the context of PSH and “*challenging, disruptive, and/or disobedient behaviors*,” the medical model could benefit from such an approach, in order to maximize the broader diagnostic perspective and not diagnose the child/adolescent based on a gap-based training culture. See Figures 1A,B below.

### Learning from Indigenous people’s ecology

As the failure in diagnosing RLS in vulnerable patients and RLS in otherwise not as vulnerable labeled children demonstrates, without considering the entire context and contributing factors, the current subspecialized urban Western diagnostic model may miss important information that can assist with the diagnosis. The failure to understand and implement these disparities is similar in context to that of a vulnerable child with PHS presenting with unexplored, thus not understood behaviors, who based on a gap-based diagnostic model is only treated with medications (17, 25, 44). In developing a shared understanding based on reciprocal communication and acknowledging the ecology of the *lived experience*, there are lessons to be learned from the ongoing discourse with Indigenous peoples (61). The recent discussions surrounding health care and delivery in Indigenous peoples are perhaps driven by different political perspectives, a colonization-induced sense of indebtedness and considerations based on political correctness. However, social ecological concepts and transdisciplinary health do not only support indebtedness and considerations based on political correctness, but also highlights the need for the necessary change and expansion of subspecialized urban Western medical models, as it offers a personalized but neutral and comprehensive discourse on individual goals and outcome measures. For ease of expression, we will refer to Canada’s Indigenous peoples (First Nations, Inuit, and Metis) and Australian Aboriginal and Torres Strait Islander populations as “*Indigenous Peoples*.” Studies in countries with a shared history of European colonization and disconnection from culture and country, (such as the United States, Canada, Australia, and New Zealand), indicate an increased risk of poor overall health and poor sleep health specifically, for Indigenous peoples compared to their non-Indigenous peers (62). Dispossession from their land, sea, country, historical colonization and interruption of culture, all of which contribute to intergenerational trauma coupled with racism and systemic inequalities have significantly impacted and disrupted Indigenous peoples’ capacity to maintain their health (63). Examination of the global literature on Indigenous perceptions of health and wellbeing, shows that concepts of overall well-being differ significantly between Indigenous and non-Indigenous populations (64).

Across Australia for example, Indigenous peoples share important spiritual and cultural beliefs that connect them to land, sea, and country with diverse cultural traditions (65) all of which contribute and are related to their physical and mental health (see Figure 1C below) often referred to as Social Emotional Wellbeing (SEWB) (58). Encompassing aspects of the physiological, psychological, environmental and cultural individual from a truly holistic perspective, is deemed as the *only way* to truly understand a problematic health presentation from an Indigenous’ perspective (64, 66). Consequently, differences exist in interactions with the Indigenous view of health versus the Western healthcare system, which are discussed here.

### The biopsychosocial and ecological perspective

The concept of acknowledging the lived experience and ecology of the community or family, respectively, as seen from the Indigenous peoples’ and/or Western perspective, is a neutral and advanced way to operationalize the necessary steps and can also support overcoming even prejudice-based perspectives in the medical model (67). If the medical model fails to integrate information from the biopsychosocial and ecological perspective, the mismatch engenders and represents key drivers of health inequity and health care delivery (64).

**Figure 2:**
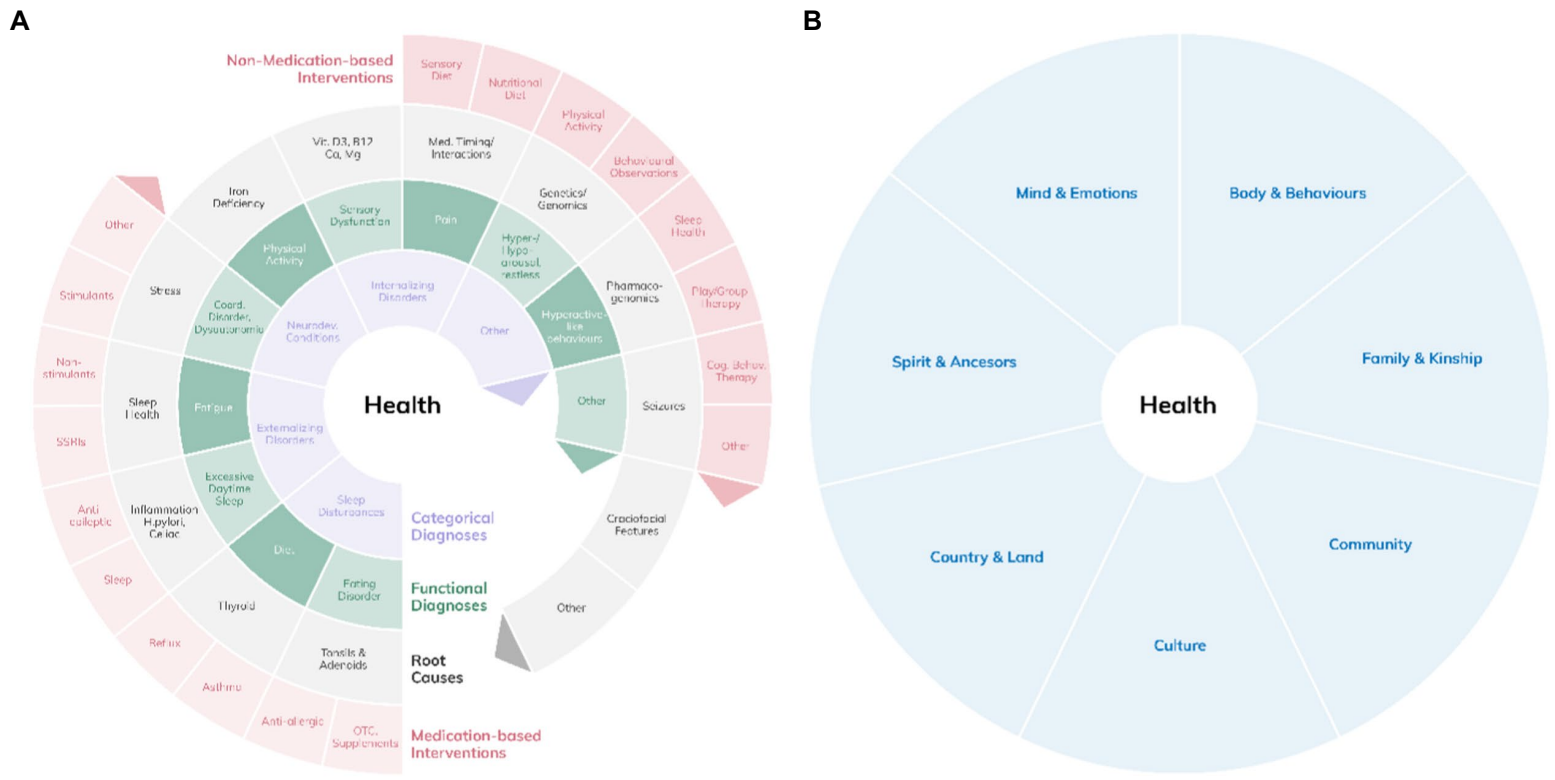
The merged Medical Logic **(A)** and SEWB models **(B)** in a transcultural, transdisciplinary and transdiagnostic screening framework. Note that the various domains of functional vs. categorical diagnoses, including sleep and wake behaviors or behavior-related diagnoses, as well as possible root causes and intervention options can be reviewed from multiple SEWB perspectives. Each perspective, e.g., “mind and emotions,” “country and land,” or “community” may change the way symptoms are perceived.

As behaviors and poor sleep health are interrelated and both are affected by our cultural background, we understand that the way to unravel the complexity of diverse perceptions and understandings in the context of sleep for people with different cultural backgrounds, is by applying the concepts of ‘exploration and observation’. Thus, we suggest the biopsychosocial model, applied through the SEWB (58) lens to become an “interface” between Western and non-Western systems or various migrant cultures of knowing. This respectful and authentic incorporation of Indigenous or migrant cultural ways of knowing, could re-inform investigations, diagnoses and interventions and integrate Indigenous epistemologies (68). Australian Indigenous peoples describe this as *“Two ways of knowing,”* while Canadian Indigenous peoples describe this as *“Two eyed seeing”* (66). As such, this model, informed by Indigenous perspectives, may be useful when working with not only Indigenous but also non-Indigenous populations. A merged model, based on an integration of the concepts discussed above is presented in Figure 2.

## Sleep health in context of diverse conceptualizations

### Examples of lived sleep experiences

Cultural assumptions about sleep form part of the patient’s ecology and have strong bidirectional relationships with vigilance and related behaviors. Thus, the awareness about sleep as the first line intervention in behavioral context, is acknowledged by multiple cultures as El Sheikh and co-authors have demonstrated in their impressive work (7, 69).

### Sleep stories

Through “sleep stories” sourced directly from a selection of Yolŋu Elders, Arnhem Land Northern Territory, Australia (70), Indigenous communities conveyed that sleep is viewed as important for health and overall well-being, but also spiritually and *via* connection to land and country and kinship (70). Yolŋu Elders recognized that “bad” sleep resulted in “bad” health and wellbeing and impacted all aspects of functioning including behavior. Fatima et al. (71) confirmed that Australian Indigenous communities view sleep and sleep health from a biopsychosocial perspective and thus very differently compared to non-Indigenous culture. Similarly, Mohawk Elders consider changes in sleep practices and sleep health as a significant disruption in children’s health and social well-being (72). In conclusion, sleep health cannot be extricated from general health or other elements that contribute to general health. Indeed, sleep disturbances in Indigenous Australian children are associated with obesity (73, 74), poorer academic performance (75), and emotional regulation and behavioral outcomes (76).

### Dreams as part of divergent conceptualizations

Dreams, often excluded from medical studies of sleep (with the exception of psychiatry) are another domain which may bring together different perspectives with an explorative approach. Among various North American Indigenous communities, such as the Dene, sleep and dreaming are both valued because they play an important role in cultural epistemology and an individual’s access to culturally valued knowledge (77). Reviewing conceptualizations, fascinatingly, Australian Indigenous peoples have also reported very different conceptualization of dreams and its impact on health (70), compared to non-Indigenous Australian families and children. While the meaning and importance of dreams is significant and related, they are different to how sleep health *per se* is seen and understood, subsequently impacting how sleep disturbances are approached in Indigenous children everywhere and have been subject not only for Australian Indigenous communities (70, 78), but universally (79) for thousands of years (7, 80). On the other hand, in Non-Indigenous contemporary psychology and Western theory of mind, dreams are rarely considered due to a range of historic-cultural reasons, such as psychology seeking to align itself with measurable medicalized outcomes and natural sciences identity (81).

### Including “lived experiences” in the medical model

Recognizing our own personal and cultural schemas and how they contribute to our individuality, all of which then dictate our perceptions and thus actions, assists in recognizing those of the “other” (82). As explained above, given the social and emotional well-being framework perceived from the Indigenous perspective, treating children from traditional Indigenous or other backgrounds (e.g., refugees) with a purely medical model would not be embracing a mutual and shared language, and neglect to explore a child’s broader ecological contributors. Learning from the Indigenous health conceptualization, we acknowledge that all vulnerable populations, such as children from migrant communities, who live or have lived as minorities in different surroundings, e.g., in industrialized countries, as trainees or workers, and similarly all authors of this concept paper, would profit from a merged medical and biopsychosocial, which considers the social ecology of sleep. Our proposed merged SEWB/medical logic models taking into account a holistic and transcultural approach is presented below in Figure 2.

### How to operationalize? The 4P factors

The operationalization of the synthesized two models to a transcultural, transdisciplinary and transdiagnostic screening framework can be conducted with four contextual questions:

1/2. *Precipitating and presenting factors* (why did this child/family present NOW to the clinician and what are they presenting with);
3. Predisposing *factors* (what broad spectrum and biopsychosocial cultural factors, including how historical and ongoing health disparities impact and predispose this child to poor sleep health and subsequent daytime dysfunction in the vigilance and behavioral domain); and
4. *Perpetuating* (what social ecological and cultural factors and are maintaining this poor sleep health and what epistemology informs this and is the lived experience taken into account; how long has this been going on, how significant is this now).

These four domains, comprehensively explored, offer a first joint transdisciplinary screening based high level understanding and allow a review of the functional or categorical diagnoses from multiple perspectives:

- The *child’s ecology*: at the individual level, physical and mental health contributors, considering the child’s temperament, understanding what purpose the behaviors serve for the child and their etiology.
- The *family unit’s ecology:* understanding family dynamics and culture, parenting styles, parent–child interactions, limit sitting capacity in order to understand how much these factors contribute to the presenting behavioral and vigilance symptomology. Factors such as parental and child mental health, disability, socio-economic status, stress levels, social support, and education all interrelate many in a bidirectional manner.
- The *society and community’s ecology:* to which the child belongs-societal expectations and understanding and exploration of childhood behavior in multiple settings, education and school systems. Community attitudes and expectations of treatment for poor vigilance, encompassing current medical model, pharmacological intervention and urgency.

In our opinion, gathering or just being aware of this collateral information ensures considerate and comprehensive exploration and understanding of the etiology of a child’s presentation; thus, not to miss systemic gaps with exclusive focus on one medical aspect of the challenge. Furthermore, it ensures diagnoses are not simply viewed through a western centric lens but with the view that the child’s behavior maybe be influenced by their specific cultural and societal expectations. For example, inattentive behavior and its relationship with performance maybe be viewed very differently in cultures where the need for performance excellence is heterogeneous. Similarly, restless sleep may not necessarily be viewed as problematic and therefore relevant to a diagnosis for some cultures other than Modern Western Societies. In Figure 2, we propose this a merged, hence holistic model.

## Pros and cons of an ecological model in sleep health

Before discussing the pros and cons, but in support of our argument, we should be aware that the definition of health has changed over time. In 1948, the World Health Organization, defined health as “*as a state of complete physical, mental and social well-being*.” After a long discourse, in 1984, this definition was changed and included “work actively” for health: *“the extent to which an individual or group is able to realize aspirations and satisfy needs and to change or cope with the environment.”* Eventually, the WHO Ottawa Charter (83) states that “*Health is created by caring for oneself and others, by being able to take decisions and have control over one’s life circumstances, and by ensuring that the society one lives in creates conditions that allow the attainment of health by all its members*.” Therefore, reviewing “*challenging, disruptive or disobedient behaviors*” in context with sleep health and vigilance as outcome measures utilizing the WHO Ottawa Charter, we needed an adaptable concept helping us to operationalize our knowledge within a broader framework. To discover from the patient’s perspective how their immediate ecology impinges on their specific health and illness concerns in their individual living setting was the starting point for the merged medical/SEWB model shown in Figure 2, rather than attempting to develop some general comprehensive competency guidelines.

### The Pros

As a response to the listed shortcomings, the merged medical/SEWB model may serve as a clinical framework guide and can be applied with flexibility to cater for diverse populations with equally diverse pools of knowledge (84) in multi-professional teams. The development of such a mutually shared agenda requires for us, health care professionals, subspecialized or without any knowledge in sleep medicine, a logical screening model, to overcome constricted perspectives and disseminate the universal parts of subspecialist knowledge to the community and create a *community health agenda* with a community voice. While it is necessary to appreciate the complex social ecology of patients, it is not necessary, or even possible in a clinical context, to have a comprehensive appreciation of all the factors that affect their health and well-being. Thus, it was important for us to develop a visualized strategy to identify and review what is relevant to the patient. For structuring the approach to the patient, allowing a reflective structure and co-creation of a therapeutic strategy (26, 44, 85), the visualization used a doubled satellite/orbit concept (86). This visualization also reverses any patient profiling, Indigenous or not, based on a presumed set of cultural traits or norms, which actually would reinforce the status quo (87). This concept highlights the interchanging dimension of affecting social emotional wellbeing factors on the medical concepts and allows integrating the patient’s perspective insightful in the center.

### The Cons

Time allocations restrict medical services and will restrict the application of theoretical concepts in everyday clinical practice. While one is expected and encouraged to consider the *whole patient*, the need for a clinical measurable outcome and immersion in the management of significant illness has reduced the ability of the treating professional to afford the time to consider the whole patient (88). Shah and Mountain suggest the medical model “*is a process whereby, informed by the best available evidence, doctors [health professionals] advise on, coordinate or deliver interventions for health improvement*” (89) (p 119). This necessitates a multidisciplinary and possibly a case management approach. We are aware of these limitations and logistical difficulties with this approach. However, we are also aware that pediatric sleep health services cannot be provided solely by urban sub-specialists and diverse disciplines have to be integrated in, e.g., developmental, mental health, and complex care teams as part of the “health” team. This is particularly the case in community settings based on a concept of tier services and the stepped care model of care (90). The synthesized wheel models (see Figure 2) highlights the necessary fluency in the medical logic model for making it functional and “integratable” into the “health” team; however, makes it difficult to use for operationalization.

## Epilogue

In this narrative, we justified why we advocate that the concept of ecology of lived experiences through a bio-psycho-sociocultural perspective should be applied in our clinical sleep medicine practice and why clinicians, who aim to diagnose, should step back and explore or screen possible contributing factors from various perspectives. In sleep medicine, structural limitations cause shortcomings and gaps in our service delivery, limiting access to this highly sub-specialized medical domain. We reviewed the shortcomings and gaps in the domain of sleep health for children and vulnerable populations, and responded to the identified challenges. Utilizing the example of “*challenging, disruptive and/or disobedient*” behaviors, we are suggesting a neutral observation-based explorative screening approach utilizing vigilance, as a reflection of poor sleep health and hyper-motor restless behaviors. The concept of *ecological systems theories* and the Indigenous “*Two ways of knowing*” and “*Two eyed seeing*,” allow a neutral framework in which one can approach different understanding and perceptions in a neutral and respectful way within these changing frameworks. This has been overdue in the context of sleep health and sleep medicine as the change in the definition and notion of the term health, as defined by WHO, mirrors the shift in our understanding and perception, opening up the discussion on community and individual cultural background. This model allows the democratization of specialty knowledge while utilizing transdiagnostic methods, as collateral information is necessary, and multiple dimensions must be considered that view child development and mental health and associated poor sleep health from a wider perspective.

## Author contributions

SB and OI conceptualized, drafted, contributed, and modeled the manuscript and developed the models. WM and TH contributed to the manuscript. All authors contributed to the article and approved the submitted version.

## Conflict of interest

The authors declare that the research was conducted in the absence of any commercial or financial relationships that could be construed as a potential conflict of interest.

## Publisher’s note

All claims expressed in this article are solely those of the authors and do not necessarily represent those of their affiliated organizations, or those of the publisher, the editors and the reviewers. Any product that may be evaluated in this article, or claim that may be made by its manufacturer, is not guaranteed or endorsed by the publisher.
